# Mechanism and Control of Meiotic DNA Double-Strand Break Formation in *S. cerevisiae*

**DOI:** 10.3389/fcell.2021.642737

**Published:** 2021-03-02

**Authors:** Vikash Kumar Yadav, Corentin Claeys Bouuaert

**Affiliations:** Louvain Institute of Biomolecular Science and Technology, Université catholique de Louvain, Louvain-La-Neuve, Belgium

**Keywords:** double-strand break, DNA recombination, meiosis, Spo11, phase separation

## Abstract

Developmentally programmed formation of DNA double-strand breaks (DSBs) by Spo11 initiates a recombination mechanism that promotes synapsis and the subsequent segregation of homologous chromosomes during meiosis. Although DSBs are induced to high levels in meiosis, their formation and repair are tightly regulated to minimize potentially dangerous consequences for genomic integrity. In *S. cerevisiae*, nine proteins participate with Spo11 in DSB formation, but their molecular functions have been challenging to define. Here, we describe our current view of the mechanism of meiotic DSB formation based on recent advances in the characterization of the structure and function of DSB proteins and discuss regulatory pathways in the light of recent models.

## Introduction

**FIGURE 1:**
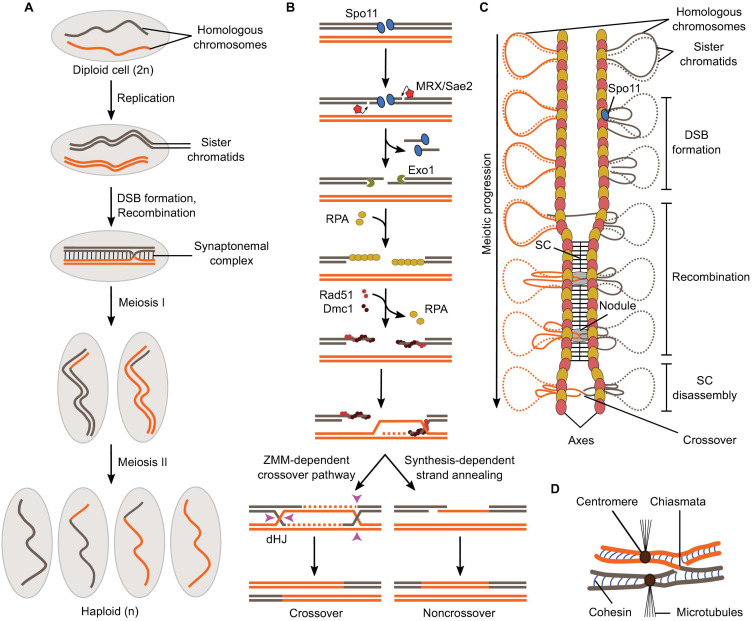
Overview of meiosis and meiotic recombination. **(A)** Schematic of the formation of haploid gametes from a diploid cell with a single pair of homologous chromosomes. DSB formation and recombination promote homolog pairing and lead to the exchange of chromosomal fragments (crossovers) in the context of synapsed chromosomes. **(B)** Meiotic recombination is initiated by Spo11-mediated DSB formation and leads to the formation of crossovers via a ZMM-dependent double Holliday Junction (dHJ) resolution pathway or non-crossovers by synthesis-dependent strand annealing. **(C)** Relationships between meiotic recombination and higher-order chromosome structure. DSB formation happens in the context of the loop-axis structure. As recombination progresses, the SC polymerizes between the axes and is disassembled prior to chromosome segregation. Axis proteins Red1 (red ovals) and Hop1 (yellow ovals) are shown. **(D)** In metaphase I, homologs are held together through chiasmata and sister chromatid cohesion.

Genomes are continuously damaged by endogenous and exogenous factors and must be accurately repaired to maintain genome integrity and function (Ceccaldi et al., 2016; Kim et al., 2016). Homologous recombination is an ancient and universal mechanism that achieves accurate repair of DNA double-strand breaks (DSBs) by copying information from an intact template (Symington, 2016; Wright et al., 2018). This repair mechanism was hijacked early during eukaryotic evolution to achieve two key goals in meiosis. First, to exchange genetic material between chromosomes, thereby breaking up allelic linkage groups and promoting genetic diversity. Second, to provide physical connections between homologous chromosomes that allow their alignment along the meiotic spindle and their accurate segregation, thereby producing chromosomally balanced haploid gametes and maintain stable genomic contents between generations (Page and Hawley, 2003; Petronczki et al., 2003; Wilkins and Holliday, 2009; Hunter, 2015; Figure 1A). Meiotic cells trigger recombination by deliberately damaging their DNA, producing hundreds of DSBs per meiosis in yeast or mice (Sun et al., 1989; Keeney, 2008; Pan et al., 2011; Kauppi et al., 2013).

Meiotic DSBs are produced by the evolutionarily conserved topoisomerase-derived protein, Spo11, along with a cohort of partner subunits (Bergerat et al., 1997; Keeney et al., 1997; Keeney, 2008; Lam and Keeney, 2015). Following break formation, Spo11 remains covalently attached to the 5′-strands at both DNA ends and is released by an endonucleolytic cleavage reaction mediated by MRX (Mre11, Rad50, and Xrs2) and Sae2, which liberates Spo11 attached to a short oligonucleotide (Neale et al., 2005; Figure 1B). The 5′-strands are further resected by 5′-3′ exonucleases (Exo1 in yeast) to produce long single-stranded tails, which are coated with ssDNA-binding protein RPA (Sun et al., 1991; Zakharyevich et al., 2010; Garcia et al., 2011; Schiller et al., 2014; Symington, 2016; Mimitou et al., 2017). RPA is then replaced by recombinases Rad51 and Dmc1 that form a nucleoprotein filament and search for sequence similarity preferentially located on the homologous chromosome, producing D-loop structures (Hong et al., 2001; San Filippo et al., 2008; Brown and Bishop, 2015). Following DNA synthesis using the homolog as a repair template, the recombination structures experience one of two main outcomes (Allers and Lichten, 2001; Hunter and Kleckner, 2001; Bishop and Zickler, 2004; De Muyt et al., 2012; Pyatnitskaya et al., 2019; Figure 1B). The invading strand can be ejected from the donor by action of helicases, which provides an opportunity for the DNA ends to re-anneal. This process is referred to as synthesis-dependent strand annealing and produces non-crossovers, that is, products not associated with reciprocal exchanges of chromosome fragments, but with local transfer of genetic information from the repair template to the broken molecule (gene conversion) (Palmer et al., 2003; Martini et al., 2011). Alternatively, recombination structures are stabilized by the “ZMM” family of proteins and channeled through a pathway that produces mostly crossovers (Börner et al., 2004; Lynn et al., 2007; Pyatnitskaya et al., 2019). Here, both ends of the break engage the donor to form a double Holliday Junction intermediate, which is resolved through a crossover-specific pathway that involves MutLγ and Exo1 (Schwacha and Kleckner, 1995; Zakharyevich et al., 2012; Gray and Cohen, 2016; Pyatnitskaya et al., 2019).

Every aspect of meiotic recombination is tied to the structural organization of the chromosomes (Figure 1C). Early in meiotic prophase, chromosomes organize as series of DNA loops that are anchored along a nucleoprotein axis. DSB formation happens in the context of this loop-axis structure. As recombination progresses, polymerization of a proteinaceous structure called the synaptonemal complex (SC) initiates between the two axes and elongates along their entire length (Kleckner, 2006; Zickler and Kleckner, 2015; Figure 1C). Recombination proceeds within the SC, inside a nodule embedded between the axes (Zickler and Kleckner, 1999). After recombination is completed, the SC disassembles and crossovers, now cytologically visible as chiasmata, provide physical connections between the homologs until their segregation at anaphase (Figure 1D).

Here, we discuss current models for meiotic DSB formation, focusing on the molecular mechanisms in *S. cerevisiae*. We present recent advances in deciphering the structure and function of proteins required for DSB formation, their interactions and relationships with chromosome organization, and discuss the mechanisms that regulate DSB formation in the light of these new models.

## Meiotic DSB Formation in *S. cerevisiae*

**FIGURE 2:**
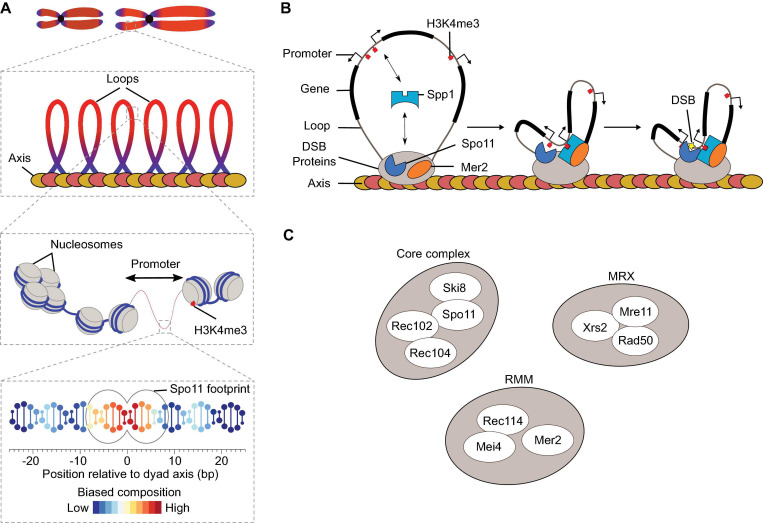
DSB formation in *S. cerevisiae*. **(A)** The distribution of meiotic DSBs is influenced by a combination of factors that operates at various size scales (Pan et al., 2011). Spo11 footprint indicates the expected occupancy of Spo11 on DNA based on structural modeling. **(B)** The tethered loop-axis model for DSB formation. Spp1 binds to H3K4me2/3 enriched around DSB hotspots and connects it to the chromosome axis through interaction with Mer2. Axis proteins Red1 (red ovals) and Hop1 (yellow ovals) are shown. **(C)** Ten DSB proteins in *S. cerevisiae*.

Meiotic DSBs are distributed non-randomly throughout the genome and concentrated within distinct regions of the chromosomes called hotspots, typically ∼50–300 base-pairs wide (Baudat and Nicolas, 1997; Petes, 2001; Buhler et al., 2007; Pan et al., 2011). The primary factor determining hotspot locations in yeast is chromatin accessibility (Baudat and Nicolas, 1997; Berchowitz et al., 2009; Pan et al., 2011). Indeed, the vast majority of the ∼3,600 *S. cerevisiae* hotspots localize within nucleosome-depleted regions at promoters (Pan et al., 2011). However, non-randomness, in terms of break distribution and intensity, can also be observed at the chromosomal scale and at the sequence level (Wu and Lichten, 1994; Lichten and Goldman, 1995; Berchowitz et al., 2009; Pan et al., 2011; Figure 2A). Indeed, chromosome size impacts DSB formation, with smaller chromosomes experiencing higher DSB densities (Pan et al., 2011; Murakami et al., 2020). DSBs are suppressed near telomeres and centromeres, and chromosomal domains with higher or lower DSB frequency alternate, correlating positively with GC content (Baudat and Nicolas, 1997; Borde et al., 1999; Gerton et al., 2000; Petes, 2001; Blat et al., 2002; Blitzblau et al., 2007; Buhler et al., 2007; Pan et al., 2011). Hotpots themselves tend to be AT-rich and are flanked by sequences enriched for the histone H3 lysine 4 trimethylation (H3K4me3) mark (Borde et al., 2009; Pan et al., 2011; Tischfield and Keeney, 2012). In addition, break formation displays sequence bias within and around the footprint of Spo11 and at the cleavage site, with a preference for cleavage 3′ of a C (Murakami and Nicolas, 2009; Pan et al., 2011; Figure 2A).

Although DSB formation happens primarily within chromatin loops, most of the DSB proteins are enriched along the chromosome axis (Kugou et al., 2009; Pan et al., 2011; Panizza et al., 2011). The tethered loop-axis model reconciles these findings by suggesting that DSB formation involves the capture of a DNA loop by axis-bound DSB proteins, allowing Spo11 to cleave the loop (Blat et al., 2002; Kleckner, 2006; Kim et al., 2010; Panizza et al., 2011; Figure 2B). The COMPASS subunit Spp1 was identified as a key player that connects the loops to the axis via interactions with H3K4me3 marks located at gene promoters and the axis-bound DSB protein, Mer2 (Acquaviva et al., 2013; Sommermeyer et al., 2013).

## The Meiotic DSB Proteins

In *S. cerevisiae*, ten proteins collaborate to form DSBs, and they can be separated into three sub-groups (Figure 2C): the core complex (Spo11, Ski8, Rec102, and Rec104), the MRX complex, and the RMM proteins (Rec114, Mei4, and Mer2) (Lam and Keeney, 2015). All ten proteins are essential for DSB formation and their deletion lead to a similar meiotic phenotype in yeast: reduced homolog pairing, loss of meiotic recombination, failure to form the SC, and abnormal chromosomal segregation ultimately resulting in inviable spores (Game et al., 1980; Malone and Esposito, 1981; Klapholz et al., 1985; Menees and Roeder, 1989; Roeder et al., 1989; Malone et al., 1991; Engebrecht et al., 1991; Cool and Malone, 1992; Galbraith and Malone, 1992; Ivanov et al., 1992; Ajimura et al., 1993; Pittman et al., 1993; Rockmill et al., 1995; Gardiner et al., 1997; Lam and Keeney, 2015). Although the molecular mechanisms whereby DSB proteins collaborate during meiosis remain unclear, recent progress has been made to understand their structure, biochemical activities and regulation. Below, we provide an overview of meiotic DSB formation emphasizing some of these recent advances.

### Spo11 and Topo VI

**FIGURE 3:**
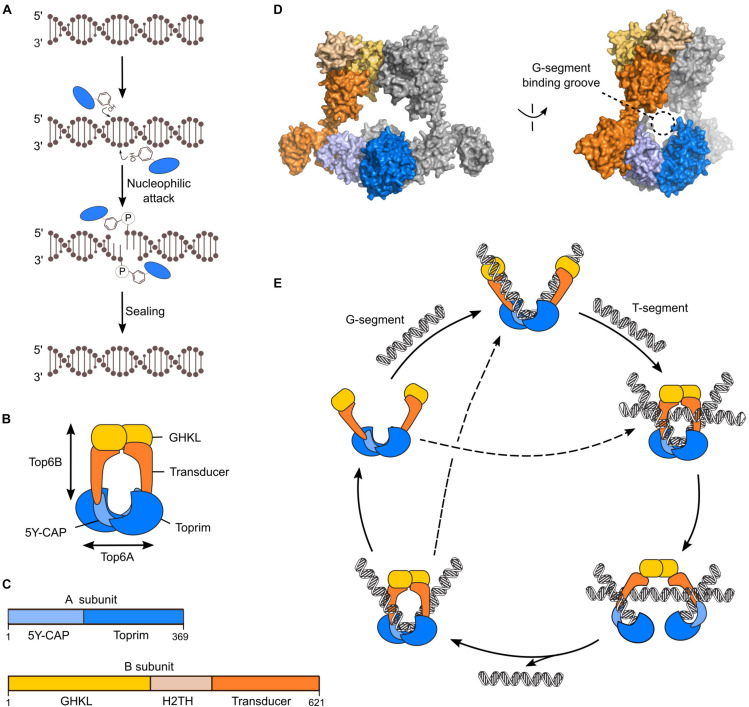
Mechanism of Topo VI. **(A)** Chemistry of strand cleavage and re-sealing in Topo VI. **(B)** Cartoon of the Topo VI heterotetramer. **(C)** Domain structure of the A and B subunits of Topo VI. **(D)** Structure of Topo VI (PDB: 2Q2E) showing the expected position of the G-segment within the groove formed by the A subunits (Corbett et al., 2007). **(E)** Catalytic cycle of Topo VI through a two-gate mechanism. ATP-dependent dimerization of the GHKL domain upon sequential or simultaneous binding to gate (G) and transfer (T) DNA duplexes is communicated to the A subunit to activate DSB formation. Topo VI can undergo multiple catalytic cycles without dissociation from the G-segment.

Spo11 evolved from the catalytic subunits of a type IIB topoisomerase, Topo VI (Bergerat et al., 1997; Keeney et al., 1997). Like other type II topoisomerases, Topo VI uses ATP binding and hydrolysis to coordinate the formation of a transient DSB to the passage of an intact duplex through the break, thereby modulating DNA topology (Corbett et al., 2007; Graille et al., 2008). Cleavage involves the coordinated action of two active-site tyrosines that attack opposite strands of the phosphoribose DNA backbone and produce 5′-phosphotyrosyl intermediates (Figure 3A). Both Topo VI and Spo11 produce staggered DSBs with 2-nucleotide 5′-overhangs (Liu et al., 1995; Buhler et al., 2001; Murakami and Nicolas, 2009). Spo11 can be thought of as a crippled topoisomerase in that it catalyzes break formation but is likely unable to perform strand passage and break re-sealing.

Topo VI has an A_2_B_2_ stoichiometry, where the A subunits perform DNA cleavage and the B subunits have ATP-binding and hydrolysis activities (Buhler et al., 2001; Corbett et al., 2007; Graille et al., 2008; Figure 3B). Although the relationship between Spo11 and Topo VIA has been recognized for over 20 years, whether Spo11 requires a B-type subunit for catalysis remained long a matter of conjecture (Bergerat et al., 1997; Buhler et al., 1998; Keeney, 2001). A few years ago, two studies eventually identified a B-type subunit in mice and plants and showed that they were essential for DSB formation (Robert et al., 2016; Vrielynck et al., 2016). This suggested that the meiotic DSB machinery is more similar to the ancestral topoisomerase than was previously appreciated. Nevertheless, while Spo11 is well-conserved and shares high sequence similarity with Topo VIA, the B-type subunits are very diverse between species and evolved almost beyond recognition from Topo VIB. Indeed, pairwise combinations of Spo11 and Topo VIA show typically 20–30% overall sequence identity with blocks that are much more conserved (Bergerat et al., 1997; Keeney et al., 1997). In contrast, conservation between the mouse Topo VIB-type subunit and *S. shibatae* Topo VIB is at best 11% identity over the most conserved 140 amino-acid block (Robert et al., 2016).

Topo VIA is composed of a 5Y-CAP domain (related to the DNA-binding domain of the catabolite activator protein) and a Toprim domain (also found in type IIA topoisomerases and in primase) (Bergerat et al., 1997; Nichols et al., 1999; Corbett et al., 2007; Graille et al., 2008; Figure 3C). Both domains participate in DNA binding and together form a groove that intimately engages the double helix (Figure 3D). The catalytic tyrosine is located in the 5Y-CAP domain and the Toprim domain coordinates Mg^2+^ ions important for catalysis. A composite active site is formed with the catalytic tyrosine and metal-ion binding pockets contributed by different subunits. Therefore, DNA cleavage necessarily requires dimerization of the A subunits. Topo VIB has an N-terminal GHKL-fold ATPase domain (found in DNA gyrase, Hsp90, Histidine Kinase, and MutL) responsible for nucleotide binding and ATP hydrolysis, a central helix two-turn helix (H2TH) fold and a C-terminal transducer domain with an extended α-helix that connects the B subunit to the 5Y-CAP domain of the A subunit (Corbett and Berger, 2003, 2005; Corbett et al., 2007; Graille et al., 2008; Figures 3C,D).

Topo VI functions through a two-gate mechanism (Corbett et al., 2007; Wendorff and Berger, 2018; Figure 3E). In its apo state, Topo VI dimerizes through the A subunits to form a U-shaped complex that can engage DNA. Topo VI binds two DNA segments, either sequentially or simultaneously (Wendorff and Berger, 2018). The G-segment (gate) binds within the DNA-binding grove formed by the A subunits and interactions between the B subunit and DNA facilitate G-segment bending (Wendorff and Berger, 2018). Engagement of the second duplex activates ATP-dependent dimerization of the GHKL domain, thereby trapping the T-segment (transfer) (Corbett et al., 2007). Dimerization of the GHKL domain is communicated to the A subunit by the transducer domain to activate DNA cleavage, whereupon ATP hydrolysis induces a conformational change that opens the DNA gate and allows strand passage (Figure 3E). Finally, the DSB is resealed, ADP in released, the ATP gate reverts to its open state, and the enzyme can dissociate from the substrate or directly engage in another round of catalysis without dissociation (Wendorff and Berger, 2018).

### The Spo11 Core Complex

S. *cerevisiae* Spo11 has long been known to closely associate with Ski8, Rec102, and Rec104 based on genetic and cytological evidence. Indeed, yeast-two-hybrid (Y2H) experiments showed strong interactions between Spo11 and Ski8, and between Rec102 and Rec104 (Arora et al., 2004; Kee et al., 2004; Maleki et al., 2007). Spo11 and Ski8 interaction is required for chromosomal localization of Rec102 and Rec104 (Arora et al., 2004; Kee et al., 2004). In addition, Rec102 and Rec104 are essential for the association of Spo11 to DSB hotspots and for Spo11 self-interaction (Prieler et al., 2005; Sasanuma et al., 2007). Furthermore, Y2H interactions with Rec114 suggested that Rec102/Rec104 may have a role to connect Spo11 with the RMM sub-group (Maleki et al., 2007).

**FIGURE 4:**
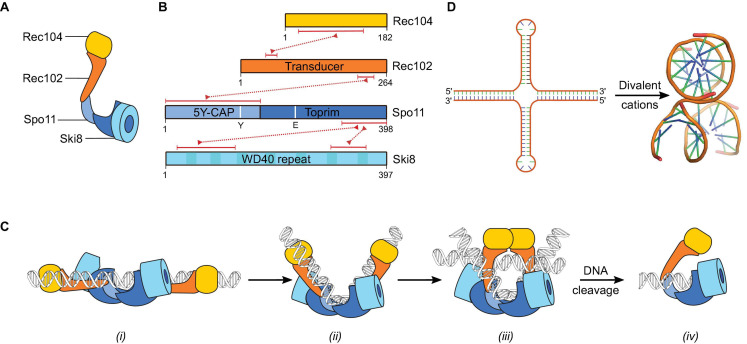
The Spo11 core complex. **(A)** Cartoon illustrating the arrangement of the different subunits in the core complex. **(B)** Domain structure of Rec104, Rec102, Spo11, and Ski8. The red dotted lines connecting two proteins represent their respective interaction domains. The region of Rec104 that interacts with Rec102 is predicted based on crosslinking-mass spectrometry, other interaction regions were validated by mutagenesis (Arora et al., 2004; Cheng et al., 2009; Claeys Bouuaert et al., 2021). The position in Spo11 of the catalytic tyrosine Y135 and metal-ion coordinating residue E233 are shown. **(C)** Proposed dynamics of the interaction between the core complex and DNA based on *in vitro* binding activities and analogy with Topo VI (Claeys Bouuaert et al., 2021). After DSB formation, Spo11 remains bound to the DSB through covalent and non-covalent interactions. **(D)** Inverted repeat sequences form cruciforms that fold into three-dimensional structures that are similar to two overlapping DNA duplexes (PDB: 1DCW) (Eichman et al., 2000).

Recent biochemical work has shown that Spo11 indeed interacts with Ski8, Rec102, and Rec104 to form a stoichiometric complex (Claeys Bouuaert et al., 2021; Figure 4A). This complex displays structural and functional similarities expected from its relationship with Topo VI, although with differences that presumably reflect their distinct biological functions (discussed below). Since DSB formation requires two Spo11 subunits and Topo VI has an A_2_B_2_ stoichiometry, the core complex was anticipated to form a dimer of tetramers. However, purified complexes turned out to have a 1:1:1:1 stoichiometry and are catalytically inactive *in vitro* (Claeys Bouuaert et al., 2021). Hence, Spo11 dimerization could be an important control mechanism for DSB formation. However, what triggers Spo11 dimerization and catalysis remains unclear.

Remote homology search had previously identified Rec102 as the Topo VIB-like subunit in *S. cerevisiae* (Robert et al., 2016). However, in contrast to the B-type subunit in mice and plants, Rec102 lacks the GHKL domain essential for ATP-dependent dimerization in Topo VI (Figure 4B). Crosslinking coupled to mass spectrometry and mutagenesis provided evidence that Rec104 occupies the position of the GHKL domain in the core complex (Claeys Bouuaert et al., 2021). Structural predictions were consistent with the possibility that Rec104 adopts a cryptic GHKL-like fold, but whether this is indeed the case needs to be confirmed. Rec104 lacks recognizable ATP-binding and hydrolysis motifs, while the B-type subunit in mice and plants retained some, but not all, the sequences thought to be important for ATP binding and hydrolysis (Robert et al., 2016; Vrielynck et al., 2016). Whether ATP is involved in meiotic break formation remains therefore unclear and it is possible that the answer differs between organisms.

In addition to Spo11 and Rec102/Rec104 that jointly form the A and B subunits derived from Topo VI, the *S. cerevisiae* core complex has an additional subunit, Ski8, with as yet unknown functions (Figure 4B). In contrast to the other core complex proteins, Ski8 is not meiosis-specific. Indeed, Ski8 has a second, independent, function as part of the Ski complex, which is involved in mRNA decay via the exosome (Anderson and Parker, 1998; Halbach et al., 2013). In vegetative cells, Ski8 localizes to the cytoplasm, but in meiotic cells it localizes to the nucleus where it interacts with Spo11 and mediates its chromosomal localization (Arora et al., 2004; Claeys Bouuaert et al., 2021). Although the meiotic function of Ski8 is conserved in *S. pombe* (Evans et al., 1997) and *Sordaria* (Tessé et al., 2003), it is not conserved in *Arabidopsis* (Jolivet et al., 2006).

Ski8 contains tandem copies of WD repeats folded into a seven-bladed β-propeller (Madrona and Wilson, 2004; Cheng et al., 2009; Figure 4B). A conserved patch of hydrophobic residues located on the top surface of the β-propeller was implicated in the interactions with Ski3 and Spo11 (Cheng et al., 2009). Indeed, the crystal structure of the Ski complex showed that Ski3 interacts with two Ski8 subunits through a sequence motif (Q–R–x–x–Φ) also found in Spo11 (Halbach et al., 2013). Mutations within this motif abolish the Y2H interaction with Spo11 and meiotic recombination and compromises the integrity of the core complex *in vitro* (Arora et al., 2004; Claeys Bouuaert et al., 2021).

Analysis of the DNA-binding properties of the *S. cerevisiae* core complex showed that the presence of divalent metal ions and the metal-ion binding residues (E233) stabilize the interactions with DNA, but the catalytic tyrosine (Y135) does not impact DNA binding (Claeys Bouuaert et al., 2021). Binding specificities directed toward different DNA structures were observed and suggested that DSB formation may be preceded by a series of conformational transitions, similar to the mechanism of Topo VI (Figure 4C). The core complex binds with low-nanomolar affinity to DNA duplexes, its anticipated DNA substrate (Figure 4C, *i*). Bound duplexes usually showed sharp ∼60° or ∼120° bends, and binding affinity was higher to pre-bent substrates than relaxed substrates, suggesting that Spo11 may bend its substrate prior to catalysis and/or bind preferentially to bendable sequences (Figure 4C, *ii*). Core complexes had particular affinity for positions where two DNA duplexes cross each other, such as plectonemic intertwinings of supercoiled DNA (Claeys Bouuaert et al., 2021). Binding to DNA junctions are reminiscent of other topoisomerases, including Topo VI (Corbett and Berger, 2005; Alonso-Sarduy et al., 2011; Wendorff and Berger, 2018), and suggest that core complexes dimerize in order to trap two duplexes (Figure 4C, *iii*). However, the stoichiometry of this intermediate was not determined and alternative interpretations remain plausible, including that monomeric core complexes have two independent duplex-binding sites. Either way, the junction-binding activity of the core complex to DNA junctions is intriguing. If the complex has more than one duplex binding site, where is the second one located? If the complex traps two duplexes like Topo VI, what is the physiological relevance of this activity, since Spo11 activity presumably does not require strand passage?

An independent line of evidence provides potential support to the hypothesis that DSB formation happens in the context of trapped DNA junctions. Insertion of long palindromes (>50 bp) within the *S. cerevisiae* genome generate meiotic DSB hotspots (Nasar et al., 2000). Palindromic sequences can extrude as cruciform structures (Benham, 1982), which are structurally similar to two duplexes crossing each other (Figure 4D). Hence, perhaps palindromes generate DSB hotspots by providing a preferred binding substrate to Spo11 and/or by inducing Spo11 catalysis through signaling that two duplexes have been captured. Similarly, human topoisomerase IIβ recognizes and cleaves DNA substrates that form four-way junctions (West and Austin, 1999).

Finally, the core complex binds with high affinity to the ends of DNA duplexes *in vitro* (Claeys Bouuaert et al., 2021; Figure 4C, *iv*). The end-binding activity was tightest with substrates that had a 2-nucleotide 5′-overhang identical to Spo11 cleavage products, suggesting that the core complex has intrinsic affinity for its product. Binding of Topo VI to the DSB intermediate has not been directly investigated, but in order for a topoisomerase to perform controlled strand passage, it must prevent swiveling of the DSB around the phosphotyrosyl bond and therefore hold on to both strands at both ends. Nevertheless, it is possible that Spo11 binds to DSB ends with much greater affinity than Topo VI. Indeed, since Spo11 does not turn over, increasing the stability of the complex from one intermediate to the next would help drive the reaction forward.

The significance of the end-binding activity is unclear, but it highlights the possibility that Spo11 binds strongly to DSBs after catalysis through covalent and non-covalent interactions. This may have implications regarding the first steps of DSB processing, since Spo11 could cap the DNA ends during resection and perhaps after strand invasion has initiated. Indeed, a recombination intermediate with Spo11-oligonucleotides capping the 3′-ends has been proposed to explain unanticipated patterns in genome-wide sequencing methods designed to map resection endpoints during meiosis in mice (Paiano et al., 2020; Yamada et al., 2020). In addition, scar-less repair by non-homologous end joining of meiotic DSBs that have undergone resection in a *Drosophila* strain with homolog pairing defects (Mcm5^A7^) provided further support for end-capping by Spo11-oligonucleotides after resection had initiated (Hatkevich et al., 2020). Nevertheless, end-capping by Spo11-oligonucleotide complexes has not been formally demonstrated.

### The MRX Complex

MRX is an evolutionarily-conserved complex that plays key functions in the maintenance of genomic integrity in somatic cells, including the recognition of DSBs, activation of the DNA-damage checkpoint, initiation of DSB resection, and telomere maintenance, in addition to essential roles during meiosis (Symington, 2016; Gnügge and Symington, 2017). In *S. cerevisiae*, MRX is essential for both the formation and processing of meiotic DSBs (Alani et al., 1990; Ivanov et al., 1992; Nairz and Klein, 1997; Keeney, 2001). The DSB-processing function of MRX depends on a single-strand endonuclease activity and a 3′-5′ exonuclease activity of Mre11 directed to the 5′-strand (Figure 1B; Paull and Gellert, 1998; Neale et al., 2005; Cannavo and Cejka, 2014). The endonuclease activity is controlled by phosphorylation of Sae2, which promotes its interaction with Rad50 (Cannavo et al., 2018). Indeed, a separation-of-function mutation of Rad50 (K81I) that supports DSB formation but blocks DSB processing abolishes the interaction with phosphorylated Sae2 (Alani et al., 1990; Cannavo et al., 2018).

While the function of MRX in processing DSBs is widely conserved, its role in promoting DSB formation has only been reported in budding yeast and *C. elegans* (Chin and Villeneuve, 2001). Indeed, MRX orthologs are not required for DSB formation in *A. thaliana* (Puizina et al., 2004) and *S. pombe* (Young et al., 2004), and whether they are required in mice remains unknown (Lam and Keeney, 2015). In *C. elegans*, MRE-11 and RAD-50 are important for DSB formation (Chin and Villeneuve, 2001; Hayashi et al., 2007), but NBS-1, the ortholog of Xrs2, is not (Girard et al., 2018).

In *S. cerevisiae*, MRX is thought to be recruited to the DSB machinery in part through interactions between Xrs2 and Mer2, based on Y2H experiments (Arora et al., 2004; Henderson et al., 2006). In addition, chromatin immunoprecipitation (ChIP) analyses show that Mre11 associates transiently to DSB sites independently of the catalytic activity of Spo11 (Borde et al., 2004). Mre11 binding to DSB hotspots requires all other DSB proteins, except Rad50, suggesting that MRX is the last component of the DSB machinery to be recruited. Perhaps MRX recruitment activates Spo11 catalysis, but how this may be achieved is unknown. It has been proposed that the requirement of the MRX complex prior to DSB formation ensures the coordination between DSB formation and subsequent repair to limit potential impacts on genomic instability (Borde et al., 2004). Indeed, breaks detected in wild-type cells are usually fully resected, indicating that they are processed faster than they accumulate, consistent with a coordination between DSB formation and repair (Bishop et al., 1992; Tran et al., 2002; Joshi et al., 2015; Mimitou et al., 2017).

**FIGURE 5:**
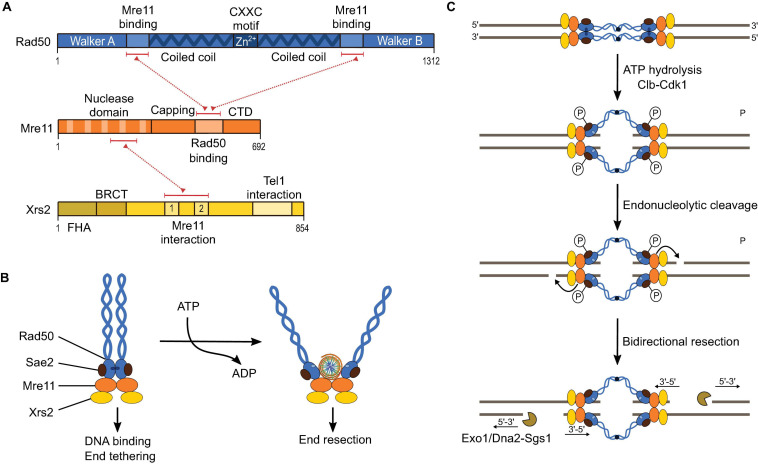
The MRX complex. **(A)** Domain structure of Mre11, Rad50, and Xrs2, and their protein-protein interacting regions (red dotted lines). **(B)** Cartoon illustrating the structural arrangement of the MRX complex and the conformational dynamics upon ATP hydrolysis. In an ATP-bound state, the nuclease domain of Mre11 does not access DNA. However, after ATP hydrolysis by Rad50, a conformational change exposes the nuclease domain of Mre11 to DNA. Sae2 is illustrated here as interacting with Rad50 based on Cannavo et al. (2018), but interactions with Xrs2 have also been demonstrated (Liang et al., 2015). **(C)** Model for DSB resection by MRX. Endonuclease activity of Mre11 directed on the 5′-strand is followed by bi-directional resection through the 3′-5′ exonuclease activity of Mre11 and the 5′-3′ exonuclease activity of ExoI or Dna2-Sgs1 in vegetative conditions or ExoI in meiosis.

Mre11 has an N-terminal nuclease domain containing five conserved phosphoesterase motifs that form the active site (Figure 5A; Arthur et al., 2004; Williams et al., 2008). Mre11 dimerizes via its phosphodiesterase domain, which is flanked by a capping domain and creates a U-shaped structure with a cleft that binds DNA and Rad50 (Figure 5B). The capping domain is followed by a Rad50-interaction domain and a C-terminal domain with DNA-binding activity (Schiller et al., 2012). The C-terminal DNA-binding domain is dispensable for mitotic DNA repair but important for meiotic DSB formation (Furuse et al., 1998; Usui et al., 1998).

Rad50 is an ATPase with Walker A and B motifs located at its N- and C-termini, respectively (Hopfner et al., 2001; Gobbini et al., 2016; Figure 5A). These are separated by a long linker that folds into a dimeric coiled-coil with the ATP-binding domain at one end and a zinc-hook domain at the other (Figure 5B; Hopfner et al., 2002; Wiltzius et al., 2005). MRX complexes can tether the two ends of a DSB via Zn^2+^-dependent dimerization of their hook domain (Hopfner et al., 2002; Hohl et al., 2010; Seifert et al., 2016; Figure 5C). Conformational changes within Rad50 upon ATP binding and hydrolysis control MRX function (Hopfner et al., 2001; Gobbini et al., 2016; Liu et al., 2016; Casari et al., 2019). In the presence of ATP, Rad50 adopts a closed dimeric conformation that occludes the nuclease domain of Mre11. Upon ATP hydrolysis, the Rad50 dimer dissociates, allowing the active site of Mre11 to access DNA (Hopfner et al., 2001; Liu et al., 2016; Casari et al., 2019; Figures 5B,C).

Xrs2 is thought to act as a molecular chaperone that connects Mre11 to other repair proteins, including Sae2 and the DNA-damage response kinase Tel1 (Oh et al., 2016). Xrs2 contains a fork-head associated (FHA) domain, a pair of BRCA1 C-terminus (BRCT) or BRCT-like domains, an Mre11-binding domain, and a Tel1-binding domain (Shima et al., 2005; Figure 5A). Xrs2 is essential for the nuclear localization of Mre11 (Tsukamoto et al., 2005). A mutation in Xrs2 that disrupts the interaction with Mre11 (K641E) abolishes its meiotic and vegetative functions (Tsukamoto et al., 2005). However, the Mre11-interaction domain alone (residues 630–662) is sufficient for Mre11 nuclear import and the DNA damage response but does not support meiotic recombination and telomere elongation. Although the FHA domain of Xrs2 was proposed to recruit Sae2 to the site of DNA damage (Liang et al., 2015), end resection remains Sae2-dependent in the absence of Xrs2 (Oh et al., 2016) and depends on interactions with Rad50 (Cannavo et al., 2018).

In vegetative cells, localization of Tel1 to the site of DNA damage is mediated by interactions between Tel1 and Xrs2 (Nakada et al., 2003; Iwasaki et al., 2016). Mutations in the Tel1-interaction motif of Xrs2 leads to DNA-damage signaling defects and short telomeres, similar to *tel1Δ* (Nakada et al., 2003). The FHA domain of Xrs2 has been shown to mediate robust Tel1 activation and to inhibit inaccurate DSB repair (Iwasaki et al., 2016). However, artificially tethering the Tel1-interaction domain of Xrs2 to an Mre11 construct containing a nuclear localization signal was sufficient for Tel1 activation (Oh et al., 2018), showing that the FHA domain was dispensable in that context.

### The RMM Proteins

Rec114, Mei4, and Mer2 (RMM) form another sub-group of functionally conserved DSB proteins with enigmatic roles at the molecular level. Although they have long been recognized as meiotic DSB proteins in yeast, the identification of their homologs across the eukaryotic kingdom has been challenging because of sequence divergence. Nevertheless, RMM homologs have now been identified in many species, including mice and humans (Kumar et al., 2010, 2018; Stanzione et al., 2016; Tessé et al., 2017; Wang et al., 2019). While Rec114 and Mei4 are meiosis-specific, Mer2 is also expressed at low levels in vegetative *S. cerevisiae* cells and shows a unique regulation. The *MER2* transcript has an intron that is only spliced efficiently during meiosis in the presence of a meiosis-specific splicing factor, Mer1 (Engebrecht et al., 1991; Nandabalan and Roeder, 1995).

Rec114, Mei4, and Mer2 localize to chromosomes in leptonema prior to DSB formation and were proposed to act as a complex based on Y2H interactions, coimmunoprecipitation, and partial foci overlap and co-dependencies (Henderson et al., 2006; Li et al., 2006; Maleki et al., 2007; Steiner et al., 2010; Miyoshi et al., 2012). Nevertheless, the existence of a stoichiometric RMM complex has never been demonstrated. In fact, their mutual dependencies are not complete, suggesting that they could exist independently. For example, chromatin binding of Rec114 and Mei4 depend on Mer2, but Mer2 foci do not depend on Rec114 and Mei4 (Maleki et al., 2007; Panizza et al., 2011).

**FIGURE 6:**
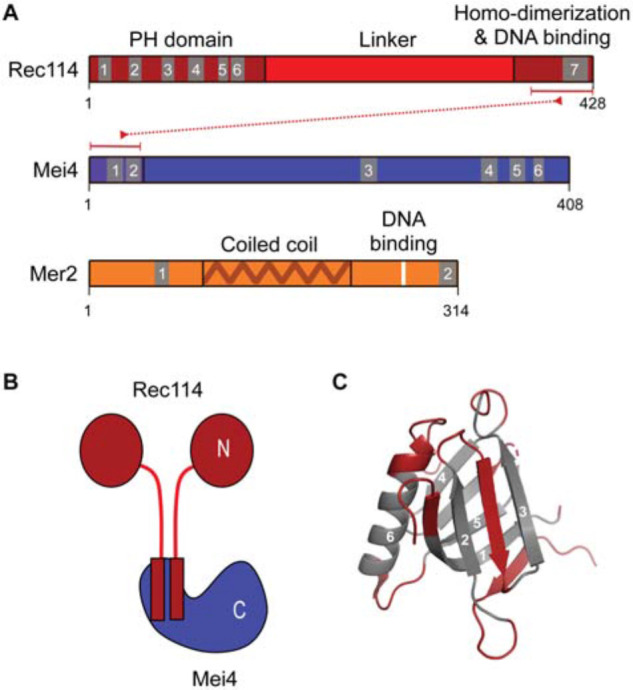
The RMM proteins. **(A)** Domain structure of Rec114, Mei4, and Mer2 with regions involved in protein-protein and protein-DNA interactions (Claeys Bouuaert et al., 2021). Numbered blocks indicate conserved sequence motifs (Kumar et al., 2010; Tessé et al., 2017). **(B)** Schematic of the Rec114—Mei4 complex. **(C)** Structure of the Pleckstrin-homology domain of mouse REC114 (PDB: 6HFG) (Kumar et al., 2018). Residues in gray are the conserved motifs highlighted in **(A)**.

Recent biochemical data revealed that the RMM proteins form two sub-complexes (Claeys Bouuaert et al., 2021). Rec114—Mei4 forms a complex with a 2:1 stoichiometry where the C-terminus of Rec114 homodimerizes and interacts with the N-terminus of Mei4 (Figures 6A,B). These sequences are amongst the most conserved regions of the proteins, suggesting that the interactions are also conserved (Kumar et al., 2010, 2018). In addition, the C-terminal domain of Rec114 is important for DNA binding by Rec114—Mei4 (Claeys Bouuaert et al., 2021). The N-terminus of *Mus musculus* REC114 was crystallized and revealed a Pleckstrin Homology (PH)-like fold with an α-helix sandwiched between two anti-parallel β-sheets (Figure 6C; Kumar et al., 2018; Boekhout et al., 2019). Blocks of amino acids previously shown to share sequence similarities across kingdoms make up the core of the domain, providing a rationale for their conservation (Maleki et al., 2007; Kumar et al., 2010). Mer2 forms a homotetramer with a predicted coiled coil thought to arrange as pairs of parallel α-helices arranged in an anti-parallel configuration (Claeys Bouuaert et al., 2021; Figure 6A). The C-terminal domain of Mer2 contains residues important for DNA binding and DSB formation (Claeys Bouuaert et al., 2021).

## Organization of the Meiotic DSB Machinery

### DSB Formation and the Chromosome Axis

It has long been appreciated that DSB formation is tied to chromosome organization (Keeney, 2001), but the relationships between local DNA-cleavage activity and higher-order structural assemblies remain poorly understood. A haploid *S. cerevisiae* genome contains approximately 700 loops, averaging about 15 kb each, with AT-rich sites that physically anchor a proteinaceous axis (Blat et al., 2002; Kleckner, 2006; Ito et al., 2014; Muller et al., 2018; Schalbetter et al., 2019). The loop-axis structure establishes in early prophase and plays important roles in DSB formation and inter-homolog repair (Carballo et al., 2008; Kim et al., 2010; Panizza et al., 2011; Zickler and Kleckner, 2015).

**FIGURE 7:**
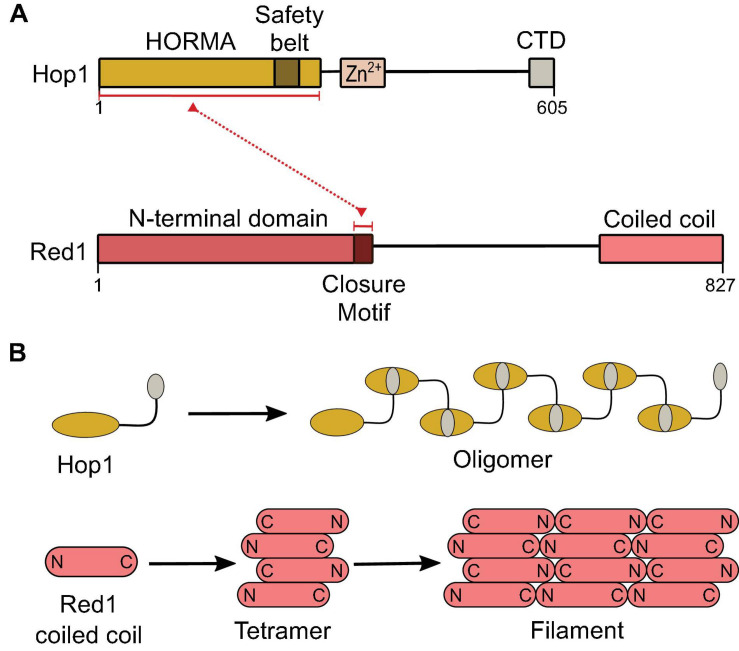
Structural components of the meiotic chromosome axis. **(A)** Domain structure of Hop1 and Red1. The C-terminal-domain (CTD) of Hop1 contains a closure motif. **(B)** Hop1 forms an oligomer through intermolecular interactions between the HORMA domain and the closure motif (West et al., 2018). The Red1 coiled-coil domain forms a parallel-antiparallel tetramer that can form a filament structure by end-to-end polymerization (West et al., 2019).

The chromosome axis in yeast includes a cohesin complex with the meiosis-specific kleisin subunit Rec8 (Klein et al., 1999), the HORMA-domain protein Hop1 (Hollingsworth et al., 1990), and the core axial protein Red1 (Smith and Roeder, 1997; Figure 7A). Axis sites are largely determined by Rec8, which localizes Red1 and Hop1 to gene ends (Panizza et al., 2011; Sun et al., 2015).

The C-terminal coiled-coil domain of Red1 forms a tetrameric parallel-antiparallel α-helical bundle (West et al., 2019). End-to-end polymerization of the coiled-coil is thought to underlie axis assembly (West et al., 2019). Red1 is thought to recruit Hop1 via its closure motif located in its central region (West et al., 2018) and Hop1 may also multimerize on the chromosome axis through head-to-tail self-assembly between the N-terminal HORMA domain and a closure motif located at its C-terminus (Kim et al., 2014; West et al., 2019; Figure 7B).

The DSB machinery is recruited to the chromosome axis prior to DSB formation. ChIP-seq experiments reveal similar DNA-binding distributions between RMM proteins and axis proteins, and chromatin association of RMM depends on axis proteins (Panizza et al., 2011; Murakami et al., 2020). Consistently, deletion of Red1 causes a 2.5- to 5-fold reduction in DSB formation and deletion of Hop1 decreases DSB levels by at least 10-fold (Woltering et al., 2000; Blat et al., 2002; Niu et al., 2005; Kugou et al., 2009). Axis proteins are therefore important for DSB formation, but their relationships with DSB proteins remain poorly understood at the molecular level.

### DNA-Dependent Condensation of RMM

**FIGURE 8:**
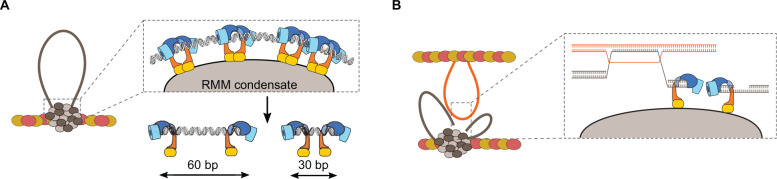
Model for the assembly of the meiotic DSB machinery. **(A)** DNA-dependent condensation of Rec114—Mei4 and Mer2 leads to the formation of large mixed nucleoprotein structures along the chromosome axis. These condensates act as a platform to recruit the Spo11 core complex, MRX, and perhaps other regulatory proteins (Claeys Bouuaert et al., 2021). This model explains the observation that Spo11 often makes closely spaced double DSBs separated with a 10-bp periodicity (Johnson et al., 2021). **(B)** Condensate-embedded core complexes may assist DNA repair by holding broken ends in the vicinity of one another. The condensates could also hold the broken chromatids through association with the base of the loops, independently of whether the DNA ends themselves are embedded. Axis proteins Red1 (red ovals) and Hop1 (yellow ovals) are shown.

Recent characterizations of the biochemical properties of *S. cerevisiae* RMM brought new insights into the relationship between DSB formation and higher-order chromatin organization. *In vitro*, Rec114—Mei4 and Mer2 complexes bind DNA with extremely high cooperativity and lead to the assembly of large nucleoprotein structures that contain hundreds or thousands of proteins, referred to as condensates (Claeys Bouuaert et al., 2021; Figures 8A, 10A). DNA-dependent clustering is therefore an intrinsic property of Rec114—Mei4 and Mer2, suggesting that it may be important for their function. Accordingly, RMM foci are cytologically visible *in vivo*, implying the local accumulation of many proteins (Claeys Bouuaert et al., 2021). Nevertheless, the biophysical nature and the composition of the foci, or their relationship with break formation, remained unclear. Evidence for a direct link between foci assembly *in vivo* and DNA-driven condensation *in vitro* came from mutagenesis approaches. Mutations within Rec114 and Mer2 with mild effects on DNA binding strongly compromised DNA-driven condensation *in vitro* and foci formation *in vivo* and abolished Spo11-dependent break formation (Claeys Bouuaert et al., 2021).

Rec114—Mei4 and Mer2 nucleoprotein condensates share properties with systems that undergo phase-separation, including the capacity to fuse upon contact and reversibility (Claeys Bouuaert et al., 2021). In the past few years, phase separation has emerged as an important mechanism that promotes self-assembly of membrane-less intracellular compartments and exerts a variety of biological functions through local enrichment of specific biomolecules (Li et al., 2012; Banani et al., 2017; Boeynaems et al., 2018). Phase separation is often driven by weak multivalent interactions involving intrinsically disordered proteins and/or RNA. In the nucleus, chromatin sub-compartments have been proposed to assemble through one of two potential mechanisms, through the self-association of a chromatin binder, or through chromatin scaffolding by a multivalent chromatin binder (Erdel and Rippe, 2018). Condensate assembly by Rec114—Mei4 and Mer2 is driven by electrostatic interactions between the negatively charged DNA and positively charged residues within RMM proteins and appears to involve a hybrid mechanism where complexes bind multiple sites simultaneously and also engage in protein-protein interactions (Claeys Bouuaert et al., 2021).

A recent study independently reported phase separation by Mer2 and its mouse homolog IHO1 (Tsai et al., 2020). In addition, Mer2 was shown to bind directly to histone octamers, suggesting the possibility that the condensates may involve chromatinized templates, not only naked DNA (Rousova et al., 2020).

Phase separation has previously been implicated in meiosis in the assembly of the SC in *C. elegans* and during homolog pairing in *S. pombe* (Rog et al., 2017; Ding et al., 2019). In *C. elegans*, interactions between SC proteins are promoted by weak hydrophobic interactions (Rog et al., 2017). This creates a SC structure with mobile constituents, which is thought to allow signal transmission at the interface between pairs of homologs and to regulate crossover distribution along chromosomes (Rog et al., 2017). In fission yeast, meiosis-specific lncRNAs-protein complexes with phase-separation properties promote robust pairing of homologous chromosomes at specific loci (Ding et al., 2019).

The biochemical properties of *S. cerevisiae* RMM suggest a model where condensates recruit Spo11 and other regulatory proteins to provide a coherent cluster for controlled DSB formation (Figure 8A). Indeed, *in vitro*, the core complex can be recruited to RMM condensates via at least two sets of interactions, one dependent on Mer2, the other dependent on contacts between the PH-fold domain of Rec114 and the Rec102—Rec104 subunits of the core complex (Claeys Bouuaert et al., 2021).

The coherence provided by the condensate could provide a mechanism to keep the broken chromatids in the vicinity of each other during repair, which may reduce the risks of gross chromosomal rearrangements. Indeed, the base of the cleaved loop would remain associated with the condensate after cleavage, and one or both ends of the DSB, capped by Spo11-oligonucleotide complexes (above), could also remain embedded within the condensate (Claeys Bouuaert et al., 2021; Figure 8B).

### Hyperlocalized Formation of Coincident DSBs

Independent evidence providing strong support for a higher-order assembly model of the DSB machinery came from the analysis of break patterning in *S. cerevisiae* (Johnson et al., 2021). Sequencing of covalently bound Spo11-DNA complexes revealed short DNA molecules (ranging from 33 to >100 bp) that are independent of MRX/Sae2-mediated nuclease activity (*sae2Δ*, *mre11nd* (nuclease dead), or *rad50S*). These arise from situations where two Spo11 complexes catalyze break formation in close proximity from one another. Double-cuts account for ∼5–20% of total Spo11 activity in wild-type cells, much higher than expected if the DSBs were independent from one another. Therefore, a mechanism must explain the formation of hyper-localized DSBs.

An important clue came from their spatial patterning, which shows a periodicity of ∼10.5 bp corresponding to the helical pitch of DNA (Johnson et al., 2021). Therefore, Spo11 complexes cutting adjacent to one another must attack the same side of the double helix. This could arise if Spo11 complexes were immobilized on a surface, prior to engaging the DNA substrate (Figure 8A). Given the DNA-dependent condensation property of the RMM proteins, axis-embedded RMM condensates are a good candidate to provide this surface (Claeys Bouuaert et al., 2021). However, whether the core complex is only recruited to the surface, or only active at the surface, remains unknown.

## Regulation of DSB Formation

**FIGURE 9:**
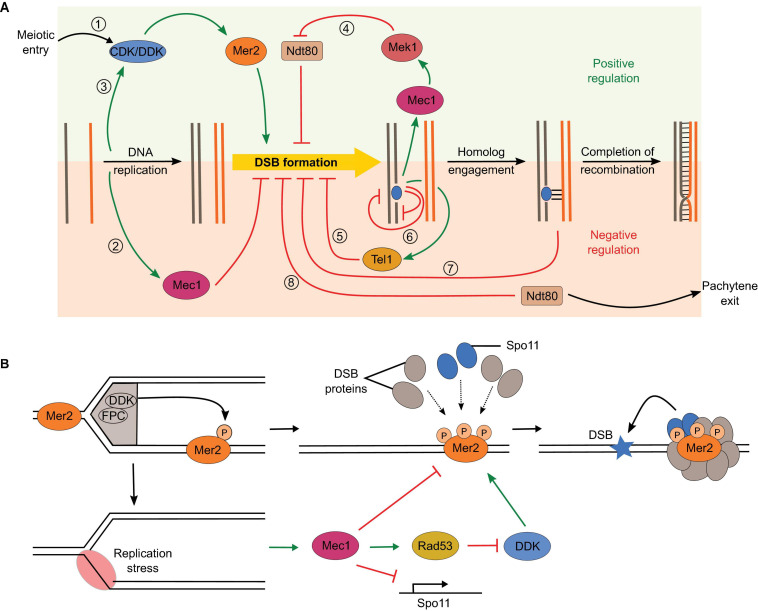
Overlapping regulatory circuits control DSB formation. **(A)** (1) DSB formation is tied to cell cycle control through dependence on CDK and DDK phosphorylation of Mer2. (2) Replication stress inhibits DSB formation by different mechanisms through activation of the Mec1 checkpoint kinase. (3) Replication also positively impacts DSB formation by promoting Mer2 phosphorylation. (4) Recombination defects activate Mec1, which extends prophase by preventing Ndt80 activation, thereby producing a positive feedback loop. (5) Activation of the DNA-damage response kinase Tel1 inhibits further DSB formation, thereby creating a negative feedback loop. (6) Hotspot competition (Tel1-independent) and DSB interference (Tel1-dependent) impact spatial distribution of DSB formation, which limits the coincident formation of two DSBs in *cis* within a 100-kb range or in *trans* between allelic regions of sister chromatids or homologs. (7) Homolog engagement shuts down DSB formation through SC-dependent removal of DSB proteins. (8) Exit of pachytene following Ndt80 activation ends the DSB-permissive period. **(B)** Positive and negative impacts of DNA replication on DSB formation. DDK is bound to the replisome via interactions with the fork protection complex (FPC). Phosphorylation of Mer2 in regions that have undergone replication promotes the assembly of the DSB machinery and DSB formation (Murakami and Keeney, 2014). However, replication stress activates Mec1 and inhibits DSB formation by reducing Spo11 transcription, inhibiting DDK via Rad53, and independently inhibiting chromatin association of several DSB proteins (Blitzblau and Hochwagen, 2013).

Since DSB formation is potentially dangerous, the activity of Spo11 is controlled to ensure appropriate timing, number, and distribution of breaks (Figure 9A). Complementary mechanisms overlap to achieve controlled DSB formation: (i) Activation of DSB formation is controlled temporally by protein expression and by coordination with the cell cycle and DNA replication through the reliance on post-translational modifications; (ii) Positive and negative feedback loops provide homeostatic control of DSB levels; (iii) Locally, DSBs distribution is controlled by a pro-active mechanism of hotspot competition and a reactive mechanism of DSB interference; (iv) Finally, the window of opportunity of DSB formation is controlled at the chromosomal scale through a recombination-dependent feedback mechanism, and globally through pachytene exit.

### Temporo-Spatial Regulation

Meiotic DSBs occur in a narrow window of time during early prophase I. This temporal regulation is achieved by a series of factors. One level of activation is implemented by meiosis-specific transcription of genes encoding DSB proteins (*SPO11, REC102*, *REC104*, *REC114*, and *MEI4*) and meiosis-specific splicing of *MER2* (Keeney, 2001, 2008). A second level is implemented through dependence of DSB formation on cell cycle progression and on coordination with DNA replication (Borde et al., 2000; Henderson et al., 2006; Wan et al., 2008; Murakami and Keeney, 2014).

S-phase cyclin-dependent kinase (CDK-S) and Dbf4-dependent kinase Cdc7 (DDK) are both essential for replication origin firing and later for DSB formation (Masai and Arai, 2002; Benjamin et al., 2003; Henderson et al., 2006; Matos et al., 2008; Wan et al., 2008). CDK-S and DDK sequentially phosphorylate Mer2 at S30 and S29, respectively, and this is important for the chromatin association of Rec114 and Mei4, and the interaction between Mer2 and Xrs2 (Henderson et al., 2006; Wan et al., 2008; Panizza et al., 2011; Figure 9A, circuit 1).

Phosphorylation of Mer2 by DDK is temporally coordinated to DNA replication by tethering of DDK to the replisome component Tof1 (Matsumoto et al., 2005; Murakami and Keeney, 2014). Mer2 phosphorylation by DDK in the wake of the replication fork therefore serves as a mark to assemble the DSB machinery in chromatin regions that have completed DNA replication (Figure 9A, circuit 3 and Figure 9B, top). However, there is a lag of about 90 min between DNA replication and DSB formation (Borde et al., 2000; Murakami and Keeney, 2014). The events that must take place between Mer2 phosphorylation and DSB formation are unclear, but in the light of the DNA-driven condensation properties of Rec114—Mei4 and Mer2, this delay could be explained by the time required to assemble the condensates and recruit the core complex and MRX.

Replication stress downregulates DSB formation through Mec1 via three complementary mechanisms: (1) partial inhibition of Spo11 transcription, (2) inhibition of DDK via Rad53 leading to hypophosphorylation of Mer2, and (3) inhibition of chromatin loading of Rec114 and Mre11 (Blitzblau and Hochwagen, 2013; Keeney et al., 2014; Figure 9A, circuit 2 and Figure 9B, bottom).

In *S. pombe*, blocking DNA replication also abolishes meiotic DSB formation (Ogino and Masai, 2006). In addition, early replicating regions are associated with higher DSB levels in *S. pombe* and in mice (Wu and Nurse, 2014; Pratto et al., 2020).

### Hotspot Competition and DSB Interference

**FIGURE 10:**
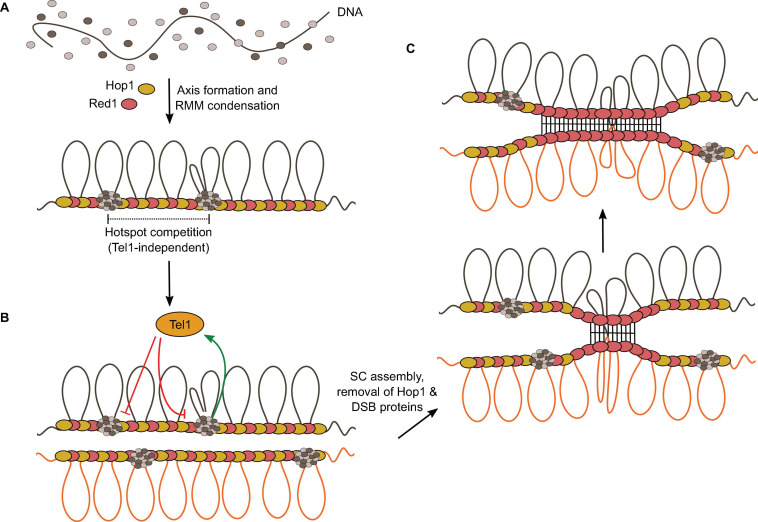
The condensate model for hotspot competition, DSB interference, and homolog engagement. **(A)** The model suggests that hotspot competition is mediated prior to DSB formation through partitioning of RMM proteins into condensates, locally depleting pools of free DSB proteins. **(B)** DSB formation activates Tel1, which inhibits local DSB formation. **(C)** SC assembly leads to the removal of Hop1 and DSB proteins from the axis, thereby shutting down further DSB formation.

DSB formation is controlled to ensure non-random distribution of recombination events along the chromosomes (Figure 9A, circuits 5 and 6). The presence of a strong hotspot suppresses the DSB activity of an adjacent hotspot (Wu and Lichten, 1994; Xu et al., 1995; Keeney et al., 2014). This phenomenon, termed hotspot competition, is observed at a population level and can be explained by a competition between hotspots for a slowly diffusing factor that is limiting for DSB formation. Hotspot competition can therefore be implemented prior to DSB formation, and RMM proteins have been suggested to constitute this limiting factor based on the fact that they are bound to the chromosome axis, which would constrain their diffusion (Panizza et al., 2011). The condensation properties of RMM proteins provide a molecular framework to understand how this may be achieved. Partitioning of Rec114—Mei4 and Mer2 complexes within condensates lead to a local depletion of free proteins, which would reduce the probability of nucleation of other condensates nearby, leading to a non-random distribution of DSB-competent zones along the chromosomes (Claeys Bouuaert et al., 2021; Figure 10A). Consistently, in *Sordaria macrospora*, the Mer2 homolog Asy2 form regularly spaced foci along the chromosome axis throughout leptotene and zygotene (Tessé et al., 2017).

Hotspot competition is genetically separable from DSB interference, the phenomenon whereby the formation of a DSB at one locus reduces the chances of another break in its vicinity. Interference is observed at the level of individual chromatids and depends on the DNA-damage response kinase Tel1, but hotspot competition does not (Mohibullah and Keeney, 2017). Upon DSB formation, Tel1 suppresses further DSB formation via a negative feedback loop thought to be implemented in part through phosphorylation of Rec114 (Zhang et al., 2011; Carballo et al., 2013; Figure 10B). Indeed, Rec114 subunit has eight [S/T]Q motifs, the known target of signal transduction kinases Tel1 and/or Mec1 (Sasanuma et al., 2007; Carballo et al., 2013), and mutation of all potential phosphorylation sites to alanine leads to elevated DSB formation, consistent with phosphorylation-dependent regulation of Rec114 (Carballo et al., 2013).

Tel1 and Mec1 mediate DSB interference in *cis* between different regions of the same molecule within about 100-kb range, and in *trans*, at allelic loci between sister chromatids (Zhang et al., 2011; Garcia et al., 2015; Fowler et al., 2018). In addition, DSB interference also occurs in *trans* between homologs, which must therefore depend on interhomolog contacts. Indeed, *trans* interference between homologs is reduced in the absence of Dmc1 (Zhang et al., 2011).

In the absence of Tel1, not only is *cis* interference abolished, but DSB formation shows negative interference within about a 10-kb range, meaning that coincident DSBs happen more often than predicted by chance (Garcia et al., 2015). Negative interference in the absence of Tel1 is explained by the condensate model of DSB formation since multiple Spo11 complexes are recruited within condensates, creating zones of high potential DSB activity that must be kept in check by Tel1 (Figure 8).

Hotspot competition and Tel1-dependent interference have been demonstrated in *S. pombe* (Fowler et al., 2018). In addition, *ATM^–/–^* mice show a high elevation of Spo11 breaks (Lange et al., 2011) and compromising ATM in *Drosophila* oocytes leads to increased levels of DNA damage (Joyce et al., 2011), showing that the Tel1/ATM-mediated negative feedback loop is conserved in mice and flies.

### Homolog Engagement

In yeast, ZMM mutants defective for synapsis and crossing over experience persistent DSB formation (Thacker et al., 2014). This revealed that excessive DSB formation is controlled by a regulatory feedback mechanism that depends on interhomolog interactions (Figure 9A, circuit 7). Yeast strains with karyotype abnormalities show accumulation of DSBs specifically on the chromosomes that experience homolog engagement defects, showing that the feedback control operates in a chromosome-autonomous fashion (Mu et al., 2020). Smaller chromosomes also experience higher DSB levels, in part because they take more time to find each other, and as a consequence remain longer in a DSB-competent state due to the persistence of DSB proteins (Murakami et al., 2020).

Mutations in components of the SC central region (Gmc2 and Ecm11) that abolish SC elongation but not crossover formation show elevated DSBs (Humphryes and Hochwagen, 2014; Voelkel-Meiman et al., 2016; Lee et al., 2020; Mu et al., 2020). This indicates that homolog engagement feedback control operates at the level of SC assembly rather than recombination.

SC assembly removes Hop1 from the chromosome axis (Börner et al., 2008; Chen et al., 2014). This is thought to close the window of opportunity for DSB formation by triggering the dissociation of DSB proteins (Mu et al., 2020; Figure 10C). Indeed, many DSB proteins (Rec102, Rec104, Rec114, and Mei4) are removed from synapsed chromosomes (Kee et al., 2004; Li et al., 2006; Maleki et al., 2007; Panizza et al., 2011; Carballo et al., 2013). In addition, chromosomal regions ∼100 kb adjacent to telomeres retain Hop1 after synapsis and experience DSB formation in pachynema (Subramanian et al., 2019). Hop1 is removed from the axis by Pch2 that probably disrupts the interaction between Hop1 and the closure motif of Red1 (Chen et al., 2014; Kim et al., 2014; West et al., 2018).

In mice, reduced SPO11 dosage leads to synaptic defects, and unsynapsed regions display elevated DSB markers (Kauppi et al., 2013). In addition, the unsynapsed portion of the X chromosome also accumulates DSBs in wild-type male mice. Similar to yeast, synapsis leads to the removal by TRIP13 of HORMAD1 and HORMAD2, and of DSB proteins REC114 and MEI4 (Wojtasz et al., 2009; Acquaviva et al., 2020). Homolog engagement feedback control therefore appears to be conserved.

### Pachytene Exit

In *S. cerevisiae*, exit from pachytene is controlled by the Ndt80 transcription factor (Xu et al., 1995). *NDT80* activation leads to the disassembly of the SC and the removal of DSB proteins, which ends the window of opportunity for DSB formation (Figure 9A, circuit 8). As a result, *ndt80* mutants accumulate more DSBs (Xu et al., 1995; Allers and Lichten, 2001; Keeney, 2001). In mutants with recombination or synapsis defects, checkpoint activation via Mec1 activates Mek1, which inhibits Ndt80 activity and leads to the extension of prophase (Figure 9A, circuit 4; Acosta et al., 2011; Gray et al., 2013; Prugar et al., 2017). Therefore, mutants that decrease Spo11 activity experience an extended window of time for DSB formation, effectively obscuring their catalytic defects. This is thought to provide homeostatic control of DSB formation.

While the negative feedback loop dependent on homolog engagement is chromosome autonomous, the Ndt80 feedback loop is nucleus-wide. The distinction was demonstrated by epistasis analysis showing that deletion of ZMM proteins in an *ndt80* mutant leads to a further increase in DSB levels (Thacker et al., 2014). Therefore, the extension of prophase and synaptic defects contribute independently to persistent DSB formation.

In *C. elegans* and *Drosophila* oocytes, suppression of crossing over on a single pair of chromosomes lead to nucleus-wide increase in the retention of DSB proteins (Carlton et al., 2006; Stamper et al., 2013) or crossover frequency (Joyce and Mckim, 2010), respectively, suggesting that recombination defects extends the DSB-permissive period, leading to global increase in DSB formation.

## Perspectives

To conclude, recent studies have brought new insights into the mechanism and regulation of meiotic DSB formation. However, our understanding of the structure, biochemical properties, and regulation of DSB proteins remains limited, and many important questions are yet to be addressed. Why DSB formation requires the collaborative action of so many proteins has been enigmatic for a long time. Our current model provides a tentative and partial response to this question by highlighting the organizational role of Rec114—Mei4 and Mer2 in the assembly of DSB-competent sites along chromosomes. As we have seen, the phase-separation model is consistent with, and explains, many long-standing observations regarding the behavior of DSB proteins. However, it also raises new questions regarding the biophysical properties of the condensates, their assembly and disassembly mechanisms, and how these might be controlled, perhaps through post-translational modifications. What are the minimal components required for DSB formation? In addition to known DSB proteins and essential phosphorylations, is something else needed to trigger Spo11 activity? What is the role of MRX? The rationale that its presence prior to break formation allows coordination with DSB repair is straightforward, but how is it recruited and how does it impact Spo11 activity? What is the relationship between DSB proteins and axis proteins? How are their spatial distributions controlled? Since Rec114—Mei4 and Mer2 bind DNA independently of axis proteins *in vitro*, why do their chromatin-association depend on the axis *in vivo*? Current models provide a molecular framework that will guide future experiments to better understand the mechanism of DSB formation.

## Author Contributions

VKY and CCB wrote the manuscript. Both authors approved the submitted version.

## Conflict of Interest

The authors declare that the research was conducted in the absence of any commercial or financial relationships that could be construed as a potential conflict of interest.
